# A pilot study on patient-related costs and factors associated with the cost of specialist palliative care in the hospital: first steps towards a patient classification system in Germany

**DOI:** 10.1186/s12962-018-0154-3

**Published:** 2018-10-16

**Authors:** Christian Becker, Reiner Leidl, Eva Schildmann, Farina Hodiamont, Claudia Bausewein

**Affiliations:** 1 Institute of Health Economics and Health Care Management, Helmholtz Zentrum München-German Research Center for Environmental Health, Ingolstädter Landstraße 1, 85758 Neuherberg, Germany; 2 Munich School of Management, Institute of Health Economics and Health Care Management & Munich Centre of Health Sciences, Ludwig-Maximilians-Universität München, Munich, Germany; 3 Department of Palliative Medicine, University Hospital, Ludwig-Maximilians-Universität München, Munich, Germany

**Keywords:** Palliative care, Resource use, Health care cost, Patient classification

## Abstract

**Background:**

Specialist palliative care in the hospital addresses a heterogeneous patient population with complex care needs. In Germany, palliative care patients are classified based on their primary diagnosis to determine reimbursement despite findings that other factors describe patient needs better. To facilitate adequate resource allocation in this setting, in Australia and in the UK important steps have been undertaken towards identifying drivers of palliative care resource use and classifying patients accordingly. We aimed to pioneer patient classification based on determinants of resource use relevant to specialist palliative care in Germany first, by calculating the patient-level cost of specialist palliative care from the hospital’s perspective, based on the recorded resource use and, subsequently, by analysing influencing factors.

**Methods:**

Cross-sectional study of consecutive patients who had an episode of specialist palliative care in Munich University Hospital between 20 June and 4 August, 2016. To accurately reflect personnel intensity of specialist palliative care, aside from administrative data, we recorded actual use of all involved health professionals’ labour time at patient level. Factors influencing episode costs were assessed using generalized linear regression and LASSO variable selection.

**Results:**

The study included 144 patients. Mean costs of specialist palliative care per palliative care unit episode were 6542€ (median: 5789€, SE: 715€) and 823€ (median: 702€, SE: 31€) per consultation episode. Based on multivariate models that considered both variables recorded at beginning and at the end of episode, we identified factors explaining episode cost including phase of illness, Karnofsky performance score, and type of discharge.

**Conclusions:**

This study is an important step towards patient classification in specialist palliative care in Germany as it provides a feasible patient-level costing method and identifies possible starting points for classification. Application to a larger sample will allow for meaningful classification of palliative patients.

## Background

Palliative care, as defined by the WHO is “an approach that improves the quality of life of patients and their families facing the problem associated with life-threatening illness, through the prevention and relief of suffering by means of early identification and impeccable assessment and treatment of pain and other problems, physical, psychosocial and spiritual” [1]. Specialist palliative care usually involves a multi-professional team, seeks to specify personal treatment goals and aims to improve the patients’ functional abilities and quality of life. Because of an ageing population, it is expected that the need for palliative care will increase in the future, especially in Germany [2–4]. It has been claimed that palliative care contributes to cost savings in the inpatient setting while in the home care setting, economic evidence was inconclusive, with further need for costing research stated for both settings [5].

In light of complex and widely varying needs, grouping clinically similar specialist palliative care patients who require a similar complexity of care is helpful for several reasons. First, this may help to understand the patients’ resource needs more comprehensively and establish a “common language” that simplifies communication among clinicians and between clinicians and administration. Furthermore, patient classifications facilitate benchmarking across settings, support quality assurance, or inform resource allocation. Patient classifications have been developed for palliative care in Australia and the UK [6–8]. They also exist in other health care fields such as inpatient care, outpatient care, and nursing home care, where different aspects are driving care, and it has long been recognized that classifications may not function adequately when used across health care settings [9].

In Germany, currently no patient classification for palliative care is available beyond diagnosis related groups (DRG). In light of evidence that primary diagnoses may not be a good predictor of resource use for palliative care, the Australian classification for palliative care, which was developed as part of a broader classification covering sub-acute and non-acute care, took into account relevant cost drivers such as phase of illness and functional status [10, 11]. Similar efforts are currently also undertaken in England [8, 12].

In order to adequately describe patients with regard to resource use and complexity, classifications need to take into account the most important determinants of resource consumption [13]. Therefore, a first step in developing such classifications is to calculate the cost per patient per episode of care from the hospital’s perspective and to analyse the factors that influence these costs. In this pilot study, we intended to establish a costing method for application in a subsequent larger study directed at deriving casemix classes for Germany. We therefore aimed to develop methods that allow calculating the cost per episode of specialist palliative care in the hospital in Germany from a hospital’s perspective. In order to assess whether collecting the required data and cost calculation is feasible in clinical practice, we applied these to a sample of patients who received specialist palliative care in a large German hospital. In addition, we aimed to analyse factors influencing the calculated costs per episode of specialist palliative care, in order to assess if factors that were deemed important in Australia also affect cost of care in Germany.

## Methods

### Study design

Cross-sectional study collecting data on patients in a specialist palliative care unit or under the care of a specialist palliative hospital support team providing palliative care consultations.

### Study setting

The Department of Palliative Medicine at Munich University Hospital comprises a 10-bedded palliative care unit with a multi-professional team comprising 1 consultant, 2 registrars, 14 nurses, and part-time social worker, physiotherapist, breathing therapist, psychologist and chaplain. Furthermore, the department runs a multi-professional hospital support team with a consultant, two registrars, and part-time social worker and psychologist, seeing palliative patients on all wards of the university hospital.

### Study population and data sources

We consecutively included all adult inpatients who had an episode of specialist palliative care at Munich University Hospital which started after the 20th of June and was completed before the 4th of August 2016.

Episodes of medical care can generally be defined as blocks “of one or more medical services received by an individual during a period of relatively continuous contact with one or more providers of service in relation to a particular medical problem or situation” [14]. Typically, episodes of palliative care aim to maintain or improve quality of life in patients who have “an active, progressive, far advanced disease with little or no prospect of cure”, by providing “multidisciplinary assessment and/or management of the physical, psychological, emotional and spiritual needs of the person and grief and bereavement process for the person and their carers/family” [15].

In this study, we differentiated two types of episodes of care because, at the study site, specialist palliative care was provided in two settings that differed in staffing and in activities performed by staff members. The first type of episode covers patients who stayed in the palliative care unit (“episodes in the palliative care unit”). The second type of episode refers to the period of time patients who stayed in other generalist wards received consultations by the palliative care hospital support team (“consultation episodes”).

The level of accuracy of patient-level costing depends on the availability of patient-level records on resource use. Cost calculation currently used to inform DRG calculation in Germany does not fully reflect patient differences in specialist palliative care resource use because the patients’ actual use of labour time is not fully recorded at patient level and not for all types of health care professionals involved. To allow for a more elaborated costing in this context, we supplemented existing records on palliative care professionals’ patient-related activity times in the Department for Palliative Medicine’s Information System Palliative Care (ISPC) by recording patient-related labour times that were not routinely documented. Newly recorded resource use included activities such as on-call physicians’ out of hours on-call labour time, the hospital support team’s travelling time inside the hospital, actual patient contact times for nurses working on the palliative care unit, and labour time spent during handovers or team meetings. Inputs for resource valuation were derived based on reports from the palliative care department’s cost centres and information on number and type of associated health care professionals. In addition, we obtained hospital-specific unit costs for selected in-house services that were routinely recorded.

Potential explanatory variables were obtained from electronic hospital records. The patients’ functional status and severity of symptoms at the beginning and at the end of an episode of care were routinely recorded. The Australia-modified Karnofsky Performance Status (AKPS) was used to assess patients’ functional status and their activities of daily living on a 10-dimensional scale. The AKPS is widely used in palliative care and in international studies as it is modified for palliative care irrespective of the setting [16]. The Integrated Palliative care Outcome Scale (IPOS) was used to assess patients’ symptoms and problems. The IPOS score gives an indication on the overall palliative care needs either assessed by patients or by professionals [17]. In addition, the patients’ responsiveness and phase of illness were assessed at the beginning and at the end of the episode of care. Using two assessments appeared justified for these characteristics, as in studies from Australia episodes of care, on average, comprised two phases of illness [11]. Corresponding to studies on patient classification from Australia, phase of illness was categorised as stable, unstable, deteriorating or terminal [18]. All patient-level information was anonymised prior to analysis and ethics approval was obtained from the responsible research ethics committee at University of Munich (Nr. 707-15, 25.1.2016).

### Cost calculation

**Fig. 1:**
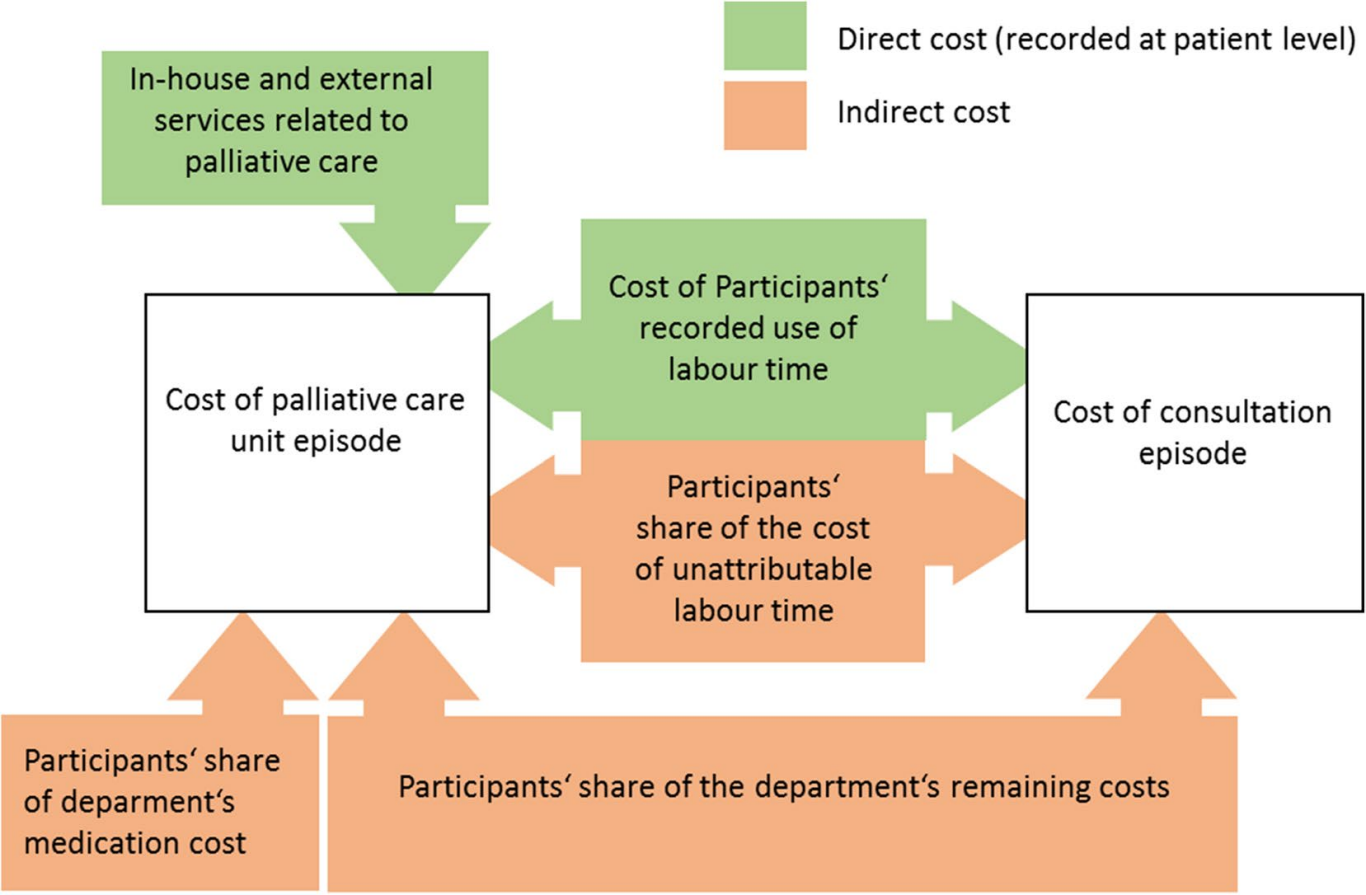
Components involved in costing episodes of inpatient specialist palliative care

We calculated the cost of specialist palliative care per episode of specialist palliative care in the hospital from the hospital’s perspective. We aimed to represent this costing perspective by considering the cost components of labour, in-house and external services, medication, and overhead cost (see Fig. 1). A focus was put on operating costs, as in Germany capital costs are publicly funded and thereby not included in patient billing [19]. According to an absorption costing approach, we allocated all incurred costs relevant to specialist palliative care to individual episodes [20]. Bottom-up costing was applied for resource use that was recorded at the patient level (direct cost). Regarding resource use that could not be attributed to individuals (indirect cost) we isolated shares of cost we assumed to be attributable to specialist palliative care and allocated these using a top-down approach. In the following sections, we present the exact costing method used for each cost category in more detail.

### Direct cost components

Palliative care professionals’ labour time that was attributable to individual patients included activities of direct hands-on care, such as talking with a patient or nursing care, as well as activities without direct contact to patients, such as patient-related administration, preparing medication, talking with relatives, consulting with other carers. The amounts of patient-attributable labour time per episode of care were determined based on electronic records and supplemental documentation by the involved health care professionals. We calculated the cost per minute of patient-attributable labour time based on the palliative care department’s total labour cost and number of occupied positions by professional group and the department’s average absence rate in the previous year. Labour costs were available for the professional groups of physicians, nurses, and allied health professionals (which include social workers, psychologists, physiotherapists, breathing therapists, and pharmacists). As seven of the breathing therapists’ 21.5 contracted labour hours per week were funded by a third party payer, their minute rate was reduced by one-third, compared to the other allied health professionals, to reflect the cost of specialist palliative care from the hospital’s perspective more accurately. Likewise, no minute rate was calculated for the chaplains’ labour time, because they were entirely funded by institutions other than the hospital. All prices were adjusted to 2016 values, based on inflation of consumer prices in Germany according to OECD [21].

The study participants’ use of services that were provided by external providers or by in-house providers other than the palliative care unit were available from electronic patient records. As no information was available on whether or not a service was part of the specialist palliative care treatment, we did not take into account this cost component in consultation episodes. In palliative care unit episodes, we considered diagnostics (laboratory tests and imaging) and in-house transportation attributable to specialist palliative care treatment. Costs of in-house and external services were calculated by multiplying the service’s resource-intensity points by the relevant internal transfer price. The available internal transfer prices were calculated according to a cost-based approach by which the relevant cost centres’ annual costs are divided by the total number of resource intensity points provided in a given year [22].

### Indirect cost components

Unattributable labour time refers to labour time that was not associated with activities that we considered patient-related. No records were available on these times, therefore, we approximated them as difference between patient-associated labour time and total labour time that we assumed had been available for palliative care in the hospital during the study period. We estimated the amount of available labour time by subtracting time devoted to activities other than palliative care in the hospital, which included estimated shares of labour time dedicated to outpatient clinic, research and teaching, and absence days from the total contracted labour time during the study period. Likewise, we accounted for unrecorded patient-attributable labour time devoted to patients who were treated in the palliative care unit during the study period but were admitted before the 20th of June or discharged after the 4th of August and therefore not part of the study population. For them, we assumed an average number of patients based on comparable data from the previous year and that average patient-attributable times for non-participants were the same as recorded for study participants. We assumed the same labour costs for unattributable labour time as for patient-attributable labour time. In agreement with previous studies, we assigned each study participant the average cost of unattributable labour time, which we calculated based on an average number of patients that was observed in the previous year [6]. Likewise, we assigned each patient a daily share of medication cost, which we calculated based on the averages from the previous year and inflation-adjustment.

Finally, we considered other overhead cost billed to the palliative care unit, which included costs related to food, medical requirements, housekeeping, maintenance, taxes, fees and insurance, and other regular expenditures, and the palliative care unit’s share of hospital overhead costs related to administration, medical and technical infrastructure, and laundry services. We extracted cost averages from the previous year from the palliative care department’s cost-centre accounting for these cost components and inflation-adjusted the costs to 2016 values. For the cost centre of “function area”, we excluded the proportion of patients at the outpatient clinic. We allocated a share of “other overhead costs” to the study participants, based on the number of days of specialist palliative care. Because the exact number of days of specialist palliative care was not recorded, we assumed that patients who stayed on the palliative care unit received specialist palliative care every day during their hospital stay. In addition, we assumed that patients with consultation episodes received specialist palliative care for a period of time that was equal to the lower bound of the time range specified by the procedure code relevant to specialist palliative care, or 1 day, if no such procedure code was specified.

### Statistical analysis

We calculated descriptive statistics on patient characteristics, patient-attributable labour time, and costs per episode. Because cost structures differed between palliative unit episodes and consultation episodes, we analysed both episode types separately.

To identify factors influencing costs per episode in palliative care unit episodes and factors influencing costs per episode in consultation episodes, we conducted multivariate analysis. To this end, we estimated generalized linear models with a gamma distribution and log-link, as these are considered appropriate to model response variables with skewed distributions, such as cost variables [23]. Independent variables were selected out of the available covariates that had fewer than 10% missing values, using the Least Absolute Shrinkage and Selection Operator (LASSO) technique [24]. Notably, length of stay, which is often used as a proxy for cost in classification studies, was not regarded as an independent variable because we considered it too closely related to the outcome variable, possibly obscuring the influence of relevant patient-level characteristics on costs [25].

## Results

This study involved 144 episodes of specialist palliative care (shown in Table 1) that mostly involved patients with primary diagnoses related to cancer. In the majority of episodes (about 80%, n = 114), patients received consultations by the hospital support team at generalist wards other than the palliative care unit, and in about 20% (n = 30) of episodes, patients stayed in the palliative care unit. On average, patients staying in the palliative care unit were 69 years old, which was 3 years older than were patients with consultations. Average IPOS and Karnofsky Scores were slightly higher at the beginning than towards the end of the episode in both episode types; however, IPOS scores were available only for a small number of participants. Frequently mentioned severe problems were worries of the family, poor mobility, and weakness. Patients were frequently in the unstable phase of illness in the beginning of palliative care unit episodes, and in the stable phase at the end. In consultation episodes, patients often remained in the same phase of illness at the beginning and at the end of an episode, mostly this was the stable phase. Patients whose phase of illness changed during a consultation episode commonly changed from stable to unstable or deteriorating phases.

**Table 1:** Descriptive statistics

Episode type	Palliative care unit (N = 30)		Consultation (N = 114)	
	n^a^	(%^a^)/mean ± SD	n^a^	(%^a^)/mean ± SD
Age	30	68.9 ± 13.5	114	66.2 ± 13.3
Sex				
Male	15	(50%)	56	(49.1%)
Female	15	(50%)	58	(50.9%)
Major diagnosis				
Oncological	25	(88.9%)	98	(87.4%)
Lung disease	–	–	1	(0.9%)
Liver disease	1	(3.7%)	3	(2.7%)
Heart disease	2	(7.4%)	8	(7.2%)
Other	–	–	2	(1.8%)
Length of hospital stay (days)	30	9.4 ± 8.2	114	6.2 ± 5.3
Episode length (days)^b^	30	9.4 ± 8.2	114	2.8 ± 3.0
AKPS at beginning of episode	30	31.7 ± 15.1	111	44.1 ± 22.1
AKPS at end of episode	30	28.0 ± 14.2	111	38.4 ± 22.1
IPOS at beginning of episode^c^	14	29.7 ± 7.1	12	25.1 ± 6.8
IPOS at end of episode^c^	13	29.2 ± 8.0	32	24.8 ± 7.6
Phase of illness at beginning of the episode				
Stable	9	(30.0%)	56	(50.5%)
Unstable	12	(40.0%)	23	(20.7%)
Deteriorating	8	(26.7%)	26	(23.4%)
Terminal	1	(3.3%)	6	(5.4%)
Phase of illness at end of episode				
Stable	12	(40%)	30	(27.0%)
Unstable	6	(20%)	32	(28.8%)
Deteriorating	7	(23.3%)	35	(34.5%)
Terminal	5	(16.7%)	14	(12.6%)
Consciousness at beginning of episode				
Alert	26	(86.7%)	61	(89.7%)
Somnolent	3	(10.0%)	3	(10.0%)
Stuporous	–	–	1	(3.3%)
Comatose	1	(3.3%)	5	(16.7%)
Consciousness at end of episode				
Alert	21	(70.0%)	85	(81.7%)
Somnolent	4	(5.9%)	8	(7.7%)
Stuporous	–	–	3	(2.9%)
Comatose	1	(3.3%)	8	(7.7%)
Number of SPC professions	30	5.8 ± 1.7	114	3.3 ± 1.7
Combinations of involved SPC professions involved				
Physician or nurse	–	–	40	(35.5%)
Physician and nurse (p&n)	–	–	6	(5.3%)
(p&n) + 1 other	4	(13.3%)	15	(13.2%)
(p&n) + 2 others	3	(10.0%)	33	(29.0%)
(p&n) + 3 others	4	(13.3%)	18	(15.8%)
(p&n) + 4 others	8	(26.7%)	1	(0.9%)
(p&n) + 5 others	5	(16.7%)	–	
(p&n) + 6 others	6	(20.0%)	–	
Type of discharge				
Home	4	(14.8%)	41	(35.7%)
Hospital	3	(11.1%)	33	(29.0%)
Nursing home	1	(3.7%)	1	(0.9%)
Hospice	6	(22.2%)	1	(0.9%)
Deceased	13	(48.2%)	24	(21.1%)
Other	–	–	14	(12.3%)

AKPS: Australian Karnofsky Performance Score; SPC: specialist palliative care; IPOS: Integrated Palliative Care Outcome Score

^a^Number/percentage of non-missing observations

^b^We defined episode length as length of stay at hospital for palliative care unit episodes and, for consultation episodes, as maximum of 1 day or the lower bound of the time range specified by the procedure code relevant to specialist palliative care

^c^Observations with no missing values for any dimension

Average length of stay was longer for patients with a palliative care unit episode, than for those with a consultation episode. Most consultation episodes involved physicians, nurses and up to three additional types of health professionals, while one third involved physicians or nurses only. Specialist palliative care professionals provided an average of 331 min of patient-attributable labour time in consultation episodes (see Table 2). Mostly, this was attributable to physician care, while breathing therapists and pharmacists had a minor role. Typical episodes at the palliative care unit involved physicians, nurses, and at least four additional health care professions. In these episodes, more than half of patient attributable labour time was provided by nurses, and about 20% by physicians and physiotherapists. In both episode types, the most time intense patient-attributable activity was direct patient care, followed by contact with other health professionals in consultation episodes and patient-related administration or preparation of medication in palliative care unit episodes. Patients with consultation episodes were often discharged home or to hospital (including intra-hospital transfer or transfer to a different hospital) and died in the hospital in about a tenth of episodes. At the palliative care unit, about half of the patients died in hospital, discharged to home or a hospice (for further nursing care) also occurred frequently.

**Table 2:** Minutes of attributable labour time by profession and type of activity

	Episode in palliative care unit (n = 30)		Consultation episode (n = 114)	
	Mean	SD	Mean	SD
Profession				
Physician	450.7	276.4	168.0	118.7
Nurse	1421.1	1200.8	78.2	90.0
Social worker	56.8	71.4	44.8	81.4
Breathing therapist	36.0	52.8	0.5	5.6
Psychologist	44.7	46.7	21.4	35.6
Physiotherapist	106.8	158.8	–	–
Pharmacist	67.3	62.7	0.1^a^	0.9
Chaplain	39.2	52.6	17.8	37.7
Total	2222.6	1771.3	330.8	242.0
Minutes by type of activity				
Direct patient care	1253.9	1112.4	107.3	107.4
Contact with relatives	250.9	237.5	55.2	68.0
Patient-related administration and preparation of medication	586.2	444.5	80.7	56.8
Contact to other carers	131.7	95.8	87.6	73.0

^a^Pharmacists are not usually involved in consultation episodes at study site

We calculated average costs of specialist palliative care of 6542€ for palliative care unit episodes and of 823€ for consultation episodes. As shown in Table 3, labour cost and other overhead cost made up about one-half of the cost per palliative care unit episode. About two-thirds of labour cost related to nursing and about one-quarter to physician care. Cost of patient-attributable labour time accounted for about half of the total labour cost. Aside from physicians and nurses, the most relevant specialist palliative care professions in terms of labour cost per palliative care unit episode were physical therapists and pharmacists. In consultation episodes (see Table 4), the cost of patient-attributable labour time, as documented in this study, accounted for about 40% of total labour cost. Most labour cost was due to physician care but health professions other than nurses and physicians, mainly social workers and psychologists were also important sources.

**Table 3:** Average cost per episode for patients who stayed at the palliative care ward (n = 30) in Euro

	Cost category	Mean	SD	Min	Max	IQR
Patient attributable (PAT) cost	PAT labour cost total	1541.7	1185.8	259.1	5462.4	1176.2
	Physician	518.3	317.9	167.1	1546.4	254.8
	Nursing	827.0	698.7	74.5	3078.9	824.6
	Other health professions total	196.5	193.5	0	837.1	226.3
	Pharmacist	44.1	41.1	0	180.2	29.5
	Breathing therapist	15.9	23.3	0	79.5	26.5
	Physiotherapist	70.0	104.0	0	533.9	91.7
	Psychologist	29.3	30.6	0	111.4	32.8
	Social worker	37.2	46.8	0	147.4	78.6
	In-house diagnostic services	92.0	174.2	0	919.1	75.3
Overhead (OH) cost	Unattributable labour cost total	1670.9	0	1670.9	1670.9	0
	Physician	257.3	0	257.3	257.3	0
	Other health professions	177.7	0	177.7	177.7	0
	Nursing cost	1235.9	0	1235.9	1235.9	0
	Medication	133.3	115.3	28.3	452.3	113.1
	Other overhead cost	3104.0	2683.9	658.1	10,529.6	2632.4
	Per episode total	6542.1	4033.5	2654.2	18,449	3579.9

**Table 4:** Average cost per episode for patients contacted by the hospital support team providing consultation (n = 114) in Euro

	Cost category	Mean	SD	Min	Max	IQR
Patient attributable (PAT) cost	PAT labour cost total	282.3	196.3	13.1	1103.5	232.9
	Physician	193.2	136.5	0	971.8	155.3
	Nursing	45.5	52.4	0	206.6	81.5
	Other health professions total	43.6	59.3	0	438.3	55.7
	Pharmacist	0.1	0.6	0	6.6	0
	Breathing therapist	0.2	2.5	0	26.5	0
	Psychologist	14.0	23.3	0	134.3	13.1
	Social worker	29.3	53.3	0	431.7	43.2
Overhead (OH) cost	Unattributable labour cost total	388.1	0	388.1	388.1	0
	Physician	257.3	0	257.3	257.3	0
	Other health professions	110.3	0	110.3	110.3	0
	Nursing cost	20.5	0	20.5	20.5	0
	Other overhead cost	152.6	167.4	55.4	775.7	332.4
	Per episode total	823.1	335.9	456.6	1937.3	519.5

The multivariate models predicting the cost per palliative care unit episode and the cost per consultation episode are presented in Tables 5 and 6. According to multivariate analysis, several covariates had a significant influence on cost per episode. In palliative care unit episodes, being in a different phase of illness in the end of the episode than in the beginning of the episode was associated with higher costs. In addition, being discharged to hospice was associated with higher costs than the other discharge types. The multivariate model for consultation episodes indicated that being male, being in a different phase of illness in the end of the episode than in the beginning of the episode, and being discharged home were associated with higher costs. A one-unit increase in Karnofsky score at the beginning of the episode was associated with slightly higher costs and a one-unit increase in Karnofsky score at the end of the episode was associated with slightly lower costs.

**Table 5:** Results of generalized linear regression with response variable “total cost per episode” for palliative care unit episodes

Parameter	exp (b)	Lower CI	Upper CI	SE	p-value
Intercept	3688.246	1686.400	8066.391	1.491	< 0.000
Age	1.001	0.993	1.010	1.004	0.755
Sex					
m vs f	0.848	0.668	1.078	1.130	0.178
Stable phase of illness (disch.)					
Yes vs no	1.235	0.953	1.600	1.141	0.111
Phase of illness change					
Yes vs no	1.552	1.184	2.034	1.148	0.001
Karnofsky (adm.)	0.995	0.983	1.006	1.006	0.350
Karnofsky (disch.)	1.007	0.995	1.019	1.006	0.243
Discharged to hospice					
Yes vs no	1.739	1.267	2.386	1.175	0.001
Deceased					
Yes vs no	0.902	0.680	1.195	1.155	0.472

**Table 6:** Results of generalized linear regression with response variable “total cost per episode” for consultation episodes

Parameter	exp (b)	Lower CI	Upper CI	SE	p-value
Intercept	1021.284	703.760	1482.069	1.209	< 0.001
Age	0.997	0.993	1.002	1.002	0.298
Sex					
m vs f	0.815	0.723	0.920	1.064	0.001
Stable phase of illness (adm.)					
Yes vs no	0.907	0.773	1.063	1.085	0.227
Deteriorating phase of illness (adm.)	1.124	0.939	1.347	1.096	0.203
Phase of illness change					
Yes vs no	1.209	1.059	1.380	1.070	0.005
Karnofsky (adm.)	1.006	1.001	1.010	1.002	0.017
Karnofsky (disch.)	0.990	0.984	0.995	1.003	< 0.001
Discharged home					
Yes vs no	1.333	1.085	1.639	1.111	0.006
Discharged to hospital					
Yes vs no	1.115	0.951	1.308	1.085	0.180

## Discussion

In this study, we piloted patient classification of palliative care patients based on their use of specialist palliative care resources by providing methods to calculate the cost of the patients’ actual consumption of specialist palliative care resources in the hospital. The average costs of 6542€ for palliative care unit episodes and 823€ for consultation episodes resulting from our calculation are the first estimates of the average cost per episode of specialist palliative care in the hospital in Germany from a hospital’s perspective. Applying the presented methods to a sample of patients in a large German hospital showed that collecting the required data is feasible in a real-life clinical setting. However, as any additional data collection requires an extra amount of work, especially over longer observation periods, strong commitment of participating institutions is required and measures such as financial incentives should be considered to improve data quality in subsequent studies. Nevertheless, studies conducted in Australia and the UK demonstrated that such data collection is possible for a certain period of time ([11]; personal communication for UK study).

The methods we outlined in this paper allow isolating the cost of specialist palliative care from other types of care that may be concomitantly provided during a patient’s hospital stay. Thereby, our unit costing approach differs from that developed by the Institute for Hospital Reimbursement (InEK) for routine use in hospitals participating in calculation of nationwide DRG cost weights in Germany, which calculates the sum of all resource use during a patient’s entire hospital stay for a given structure of cost centre and cost type [26]. Moreover, by bottom-up costing also physician labour time and selected activity times that are not routinely tracked at patient level, our method appears suitable to reflect patient heterogeneity regarding labour cost to a higher degree than previous approaches. This is highly relevant as, according to our results and previous studies, labour cost is an important cost component of palliative care [27, 28]. In contrast, the InEK’s method considers routinely documented nursing times only and allocates physician labour cost based on the patients’ number of care days. Also, studies on patient classification for palliative care in Australia did not consider the cost of physician care in their class-finding dataset [6, 11]. Our approach of bottom-up costing patient-attributable labour time and allocating equal shares of the cost of unattributable labour time corresponds to the approach taken in Australia [6]. However, as only patient related labour time was recorded, we approximated unattributable times by subtracting the recorded patient-related labour time from the total labour time that we assumed was available according to working contracts. One alternative approach would be to allocate labour costs based on a patients’ share of total patient-attributable labour time, comparable to InEK’s approach of allocating the cost of nursing care [26]. However, separating patient-related and unrelated costs reflects the patients’ actual use of labour time, whereas the latter approach amplifies differences between patients regarding the use of labour time. While total labour cost was the largest cost component, there was a large share of unattributable labour time. This could be explained by our focus on the narrowly defined activities for which we recorded activity times. Unattributable activities include, for example, standby times, breaks or walking time in between activities, and general administrative activities. Another important cost component in our calculation was the department’s overhead cost, which we included especially to reflect the patients’ use of hospital infrastructure.

To account for structural differences in palliative care provision at the study site, we calculated costs separately for two episode types. Notably, in episodes at the palliative care unit, specialist palliative care is the primary reason for a patient’s hospital stay, hence, aside from specialist palliative care in a narrower sense, a substantial amount of these episodes’ costs are related to activities such as nursing or physiotherapy. In contrast, in consultation episodes specialist palliative care consisting primarily in consultations is provided to patients as an add-on to the care they receive at different generalist wards. Therefore, from the hospital’s perspective, our cost estimate for specialist palliative care in consultation episodes should be seen as incremental cost of providing specialist palliative care in addition to the underlying acute care.

Multivariate analysis provides first insights on possible cost drivers in specialist palliative care in the hospital. However, given the small sample size these results must be seen as explorative. Notably, our models differ from existing classification approaches as they sought to explain episode costs using both variables that were collected at the beginning and at the end of an episode. While variables collected at baseline could be seen as indicators of expected resource use, those collected at the end of episode, especially discharge type, could be seen as outcome and thus, the provided treatment input. Considering both types of variables may provide relevant input in designing classifications in line with established policy objectives in palliative care [29]. As expected based on the studies from Australia, type of diagnosis was not selected for inclusion in our multivariate model explaining cost per episode. In contrast, our data showed that information on phase of illness could have an influence on cost per episode. This variable was found to be an important determinant of health care resource use in Australia as the local patient classification’s specifically takes into account each phase of illness [11]. Yet, our study’s unit of observation was the episode of care, whereas in Australian it was phase of illness. Thereby, our data does not permit unravelling which fraction of costs was incurred while the patient was in a particular phase of illness, which may be an important reason why we did not find more pronounced effects of phase of illness. A further variable that was considered in classifying palliative care patients in Australia was RUG-ADL, which quantifies the level of support patients require in activities of daily living [11]. Likewise, a recent review found indicators of functional status to improve predictive power of casemix systems, particularly for elderly or severely functioning-impaired populations [13]. Results from our study indicating that Karnofsky score at the beginning and at end of consultation episodes had an influence on episode cost point in this direction. While statistical considerations play an important role in shaping a patient classification, variables need to be examined regarding their clinical meaningfulness and, especially for classifications that are directed at informing reimbursement, with regard to the involved economic incentives.

Although our study provides an important first step towards developing a patient classification there are several limitations. Given the relatively small sample size, results of the cost calculation and multivariate analysis are not fully generalizable—constructing a patient classification that is sufficiently accurate to guide reimbursement, requires a much larger and representative sample. Practical application of our method to cost management would necessitate a comprehensive and routinely implemented collection of patient-level cost and activity data, especially with regard to labour time. In this study, by recording patient-attributable labour times, we adopted a level of detail for data collection that allowed for an adequate trade-off between accuracy and documentation effort, piloting application in a larger scale multicentre study. A more precise cost calculation could be achieved by comprehensively recording all labour time, including the amounts of unattributable labour time, the amounts of labour time dedicated to activities other than specialist palliative care (research and teaching or treatment of outpatients), and the estimated amounts of patient-related labour time provided to non-participating palliative care patients. However, this documentation effort would require substantial amount of additional work, especially over longer observation periods.

Owing to the available cost data, the degree of cause-fairness of patient-level costing was lower for particular cost components. We isolated the fraction of overhead cost relevant to specialist palliative care based on proportions of patient groups in the previous year, which is a commonly used approach in hospital cost accounting. We allocated these costs based on days of care, which is an allocation key frequently applied in cost unit accounting in hospitals [26]. As we split the overhead costs that were documented for the palliative care unit’s cost centre among the palliative care unit episodes only and the overhead cost that were recorded for the hospital support team’s cost centre among consultation episodes only, the palliative care unit episodes’ share of overhead cost is relatively high. More accuracy could be reached by further differentiating relevant patient groups for particular types of overhead cost, which could result in a higher share of palliative care unit’s cost centre’s overhead cost being allocated also to consultation episodes, but this was not possible in this study, given the available data. With regard to medication costs, only the palliative care unit’s total medication cost was available from the hospital’s accounting department. We allocated each patient a share of this cost, based on his or her length of stay in the palliative care unit. A similar procedure is also applied in DRG calculation for medication costs that are not recorded on patient level. To allow for a more precise calculation of patient-level costs, in a subsequent larger study, recording patient-level resource use should be considered.

Although documenting patient-related labour times worked well in this study, the quality of some routinely available patient characteristics that were considered as potential explanatory variables was lower than expected. Consciousness was frequently not recorded for the beginning of consultation episodes. This is probably more related to the documentation software as the item can be missed easily rather than to a clinical issue. Also, with regard to IPOS score, there was a relatively high number of patients for whom at least some dimensions of the IPOS score were available, while others were, probably because staff felt that they could not judge individual items as the patients were unconscious or imminently dying. However, for consistency, we calculated total IPOS score only if all 10 dimensions were present. While this approach increases the numbers of missing values, it increases validity and is commonly used in studies using patient-related outcome measures, such as the EQ-5D. In future studies, standard protocols for data entry should be followed carefully and quality should be closely monitored not only for cost calculation inputs, but also for potential explanatory variables.

Finally, further potentially relevant variables could be taken into account in predicting resource use. For example, a Japanese study found palliative prognostic index and necessity of a feeding device to determine whether patients were discharged home and another study found that resource use was higher if friends, family members or other kind of relatives existed [30, 31]. In multivariate analysis, we specified an indicator variable on whether patients died during their hospital stay but this was not significant for both episode types. In contrast, change in phase of illness was associated with cost per episode. Phase of illness was also identified as most relevant predictor of resource use in palliative care in Australia (a study with 5000 episodes of care), where it is currently used in classifying palliative care patients. Similar predictors were identified in a similar study just being completed in the UK (2500 episodes of care; personal communication). It is conceivable that the mentioned prognostic score reflects some of the information that is already included in the “phase of illness”—variable as people with a similar prognosis may likely be in the same phase of illness.

## Conclusions

In this study, we piloted costing episodes of specialist palliative care in the hospital in Germany based on patient-level records and routine data in a way that retained inter-patient variability in health care use, especially with regard to physician, nurse, and other health professional labour time, as a first step towards deriving a patient classification for palliative care. Applying our method to a sample of adult inpatients receiving specialist palliative care showed to be feasible in clinical practice, and thus appears suitable for application in a subsequent larger multicentre study. Although this study’s cost estimates are based on a relatively small sample and, in this stage, may not be fully generalizable, they provide first insights into factors that drive patient level costs of specialist palliative care in the hospital in Germany. Based on our methods, inputs for a classification of clinically similar patients with similar resource use could be derived from a larger sample, using statistical classification techniques.

## Abbreviations

AKPS: Australia-modified Karnofsky Performance Status
DRG: diagnosis-related groups
InEK: Institute for Hospital Reimbursement
IPOS: Integrated Palliative care Outcome Scale
LASSO: Least Absolute Shrinkage and Selection Operator
OECD: Organisation for Economic Development
UK: United Kingdom
WHO: World Health Organisation
